# Application of Poly-L-Lysine for Tailoring Graphene Oxide Mediated Contact Formation between Lithium Titanium Oxide LTO Surfaces for Batteries

**DOI:** 10.3390/polym14112150

**Published:** 2022-05-25

**Authors:** Ignacio Borge-Durán, Ilya Grinberg, José Roberto Vega-Baudrit, Minh Tri Nguyen, Marta Pereira-Pinheiro, Karsten Thiel, Paul-Ludwig Michael Noeske, Klaus Rischka, Yendry Regina Corrales-Ureña

**Affiliations:** 1 Chemistry Department, Bar-Ilan University, Ramat-Gan 5290002, Israel; ilya.grinberg@biu.ac.il; 2 National Laboratory of Nanotechnology LANOTEC, National Center of High Technology (CeNAT-CONARE), 1174-1200, Calle Costa Rica, Pavas, San José 10109, Costa Rica; jvegab@gmail.com; 3 Laboratorio de Polímeros (POLIUNA), Universidad Nacional, Avenida 1, Calle 9 Heredia 86 Heredia, Heredia 40101, Costa Rica; 4 Adolphe Merkle Institute, University of Fribourg, Chemin des Verdiers 4, 1700 Fribourg, Switzerland; minhtri.nguyen@unifr.ch; 5 Adhesive Bonding Technology and Surfaces, Fraunhofer Institute for Manufacturing Technology and Advanced Materials IFAM, Wiener Straße 12, 28359 Bremen, Germany; marta.p.pinheiro@hotmail.com (M.P.); karsten.thiel@ifam.fraunhofer.de (K.T.); michael.noeske@ifam.fraunhofer.de (P.-L.M.N.); klaus.rischka@ifam.fraunhofer.de (K.R.); 6 Faculty of Production Engineering, University of Bremen, Am Fallturm 1, 28359 Bremen, Germany

**Keywords:** density functional theory, molecular interface design, electrode, anode, polypeptide interfactant

## Abstract

When producing stable electrodes, polymeric binders are highly functional materials that are effective in dispersing lithium-based oxides such as Li_4_Ti_5_O_12_ (LTO) and carbon-based materials and establishing the conductivity of the multiphase composites. Nowadays, binders such as polyvinylidene fluoride (PVDF) are used, requiring dedicated recycling strategies due to their low biodegradability and use of toxic solvents to dissolve it. Better structuring of the carbon layers and a low amount of binder could reduce the number of inactive materials in the electrode. In this study, we use computational and experimental methods to explore the use of the poly amino acid poly-L-lysine (PLL) as a novel biodegradable binder that is placed directly between nanostructured LTO and reduced graphene oxide. Density functional theory (DFT) calculations allowed us to determine that the (111) surface is the most stable LTO surface exposed to lysine. We performed Kubo–Greenwood electrical conductivity (KGEC) calculations to determine the electrical conductivity values for the hybrid LTO–lysine–rGO system. We found that the presence of the lysine-based binder at the interface increased the conductivity of the interface by four-fold relative to LTO–rGO in a lysine monolayer configuration, while two-stack lysine molecules resulted in 0.3-fold (in the plane orientation) and 0.26-fold (out of plane orientation) increases. These outcomes suggest that monolayers of lysine would specifically favor the conductivity. Experimentally, the assembly of graphene oxide on poly-L-lysine-TiO_2_ with sputter-deposited titania as a smooth and hydrophilic model substrate was investigated using a layer-by-layer (LBL) approach to realize the required composite morphology. Characterization techniques such as X-ray photoelectron spectroscopy (XPS), atomic force microscopy (AFM), Kelvin probe force microscopy (KPFM), scanning electron microscopy (SEM) were used to characterize the formed layers. Our experimental results show that thin layers of rGO were assembled on the TiO_2_ using PLL. Furthermore, the PLL adsorbates decrease the work function difference between the rGO- and the non-rGO-coated surface and increased the specific discharge capacity of the LTO–rGO composite material. Further experimental studies are necessary to determine the influence of the PLL for aspects such as the solid electrolyte interface, dendrite formation, and crack formation.

## 1. Introduction

To achieve the goals of international environmental agreements, it is necessary to realize lower environmental footprints by designing more sustainable processes and materials for new technologies. Nowadays, technological advances of global interest are necessary to boost the use of new biodegradable, lighter, non-toxic, and recyclable materials that are also efficient and can meet market demands. Tailoring composite materials to achieve an outstanding performance throughout their usage and maintenance from the beginning of their life until a sustainable end-of-life scenario is a challenge that requires interdisciplinary mastery of multiple pieces of information and material interfaces.

Our economy has an increasing demand for rechargeable energy storage devices that facilitate fast charging rates, smaller dimensions, lighter materials, and lower leakage rates [1]. New 2D materials such as graphene, MXenes, hematenes, and transition-metal dichalcogenides are good candidates for producing more efficient solar cells, batteries, and flexible electronics [2]. In combination with polymers and further customizable particulate fillers, these 2D materials facilitate the three-dimensional tailoring of versatile composites that owe their functionality to precisely shaping the respective high specific interface areas. The advances in nanotechnology and improvements in the production of nanostructured metal oxide materials open promising directions for finding new approaches to control the carbon coating formation in the nanoscale range towards the tailoring of percolation paths. Uniform and thin carbon surface coatings on state-of-the-art oxide nano and micro particles and nano-porous structures such as aerogels are highly promising for ensuring effective electronic transport as well as maximizing the efficiency of ion insertion and extraction without limiting ion surface and bulk diffusivity [3,4,5].

Flake-shaped graphene is the most promising carbon source due to its superior electronic conductivity and excellent thermal and mechanical properties. However, graphene attaches weakly to metal oxide surfaces’ LTO and shows a tendency to form agglomerates arising from Van der Waals interactions between neighboring flakes [6]. LTO-attached binder polymers must be able to provide a stable composite material that exhibits sufficient electric transport [7]. The LTO can be used as a cathode and anode material [8], but in this research will be tested as an anode material. Binders are used to form a conductive bridge based on either physical or chemical interactions with the filler particles. The commonly used binders, such as PVDF, are electronically insulating and hamper controlling both the graphene distribution and stacking or layer thickness [9]. In general, the electrode is prepared mixing the LTO particles, the binder, and the carbon black to form a slurry that will be used for coating the current collect, and then a process such as calendaring will be applied [10]. 

Strategies for forming LTO–graphene composites mixtures such as *in situ* supramolecular self-assembly have been reported [3]. However, the processes underlying such self-assembly cannot yet provide good control of the layer thickness and morphology of the rGO layers. Layer-by-layer (LbL) assembly is used for the formation of sandwich-like films on flat substrates and offers a highly versatile method for the fabrication of controlled layered structures of different component materials (e.g., polymers, colloids, biomolecules, cells) using simple and inexpensive procedures. It is based on the sequential build-up of thin films with nanometer-level control over film thickness, which also allows precise control over the physicochemical properties of the nanostructured materials [11]. In this study, we propose to use the LbL approach, adsorbing layers of oppositely charged materials sequentially to achieve control over the layer film thickness and morphology. Non-covalent functionalization is generally preferred since it allows the introduction of new chemical groups without compromising the structural properties of graphene-type flakes, and at the same time avoids the use of aggressive chemicals and high temperatures in manufacturing [12].

Biomolecules such as proteins and DNA have evolved over billions of years into molecules capable of precise interactions and reactions, including highly specific substrate recognition, analyte binding, and directional electron tunneling [13,14]. They are promising molecular junctions for applications such as energy storage devices because they can attach to abiotic surfaces, such as metal and oxide graphene materials, enable solid-state electron transport depending on their assembly [15], and also endow the interfaces with other properties such as force response and self-healing [16,17]. They can adsorb irreversibly on surfaces, assemble in monolayers or multilayers, and form smart materials with different functions and properties [18,19]. Therefore, biomolecules can either form doping, passivation surface layers or act as a precursor due to their metal chelation properties before thermal treatment and eliminate defects at the interface to modulate the electrical and optoelectronic characteristics. Furthermore, they can adsorb ions [20], contribute to the promotion of the dissociation of lithium salts, help to wet the surface with the electrolyte, but also expose surface functionalized groups that may or may not cause Faradaic reactions [21]. The conductivity of the protein layer is related to its assembly. 

Proteins such as sericin and soy proteins have been reported as water-soluble binders and have attracted attention owing to their nontoxicity, low cost, and environmental friendliness [22].

Ryou et al. demonstrated that cross-linked alginate and catechol used as binders exhibited improved cycling performances compared to Si-PVDF [23]. Ramkumar et al. tested chitosan-glutaraldehyde crosslinked ultrathin cobalt molybdate nanosheets mixed with carbon as a cathode material for the hybrid capacitor in an aqueous environment and their results indicated an excellent cycling stability. They suggested that the ion adsorption and excellent adhesion to CoMoO_4_ enhanced the capacitive behavior [24]. Barguesi et al. showed that chitosan can be used to produce electrodes for Na ion cells without organic solvents, opening up possibilities for decreasing the production costs for building the cells [25]. The use of biopolymers as interfacial binders has been studied, but the opportunities to control graphene and other 2D materials have not yet been exploited. Stamboroski and co-workers have recently suggested the term polymeric interfactant layer for polymeric monolayers formed by amphiphilic polymers such as polypeptides or proteins [26] on various solid substrates and facilitating the attachment of further layers, for example, graphene oxide (GO) [27]. Potentially, the waterborne biomolecules used to bind these sandwich-like layer stacks can be subsequently dried and calcinated, providing a functional and sustainable carbonaceous source for tightly connecting LTO with GO that is reduced to reduced graphene oxide (rGO) with a significantly increased electronic conductivity during the same process.

Recently, we investigated a versatile and substrate-independent approach for fabricating graphene multi-layers comprising a finite vertical sequence of laterally overlapping flakes on oxide surfaces. The process allowed for manufacturing homogeneous, thin, and partially reduced rGO films (≈5 nm) in pre-determined surface topographies and thicknesses, forming a multilayer sandwich-like composite with electrically conductive hybrid graphene/protein layers on silica or semiconducting material surfaces [28]. In the present study, we describe the use of PLL for the LbL assembly of GO deposition on titanium oxide-based surfaces from a computational and experimental perspective.

Simulations based on density functional theory (DFT) provide an outstanding tool to understand and explore the role of interfaces in lithium-ion (Li-ion) battery applications and their impact on transport properties. For example, Pignanelli et al. studied Li-ion dissociation and transport in poly (acrylonitrile) host material promoted by the addition of hydrogen titanate nanotube fillers for solid polymer electrolytes by ab-initio molecular dynamics (AIMD) [29]. Additionally, the interfaces of graphene-based anodes were explored by first-principles calculations to study the effects of defects in graphene used for Li-ion batteries, and the findings revealed that where these defects combined with doped graphene may reduce the diffusion barrier [30]. Sun et al. studied the transfer mechanism of lithium ions on the surface of MnO_2_ nanosheets by performing DFT calculations to analyze the interactions between polyethylene oxide (PEO) and MnO_2_ and the relevant lithium transport that gives rise to the easy desorption of Li from PEO and migration on the MnO_2_ nanosheet. It was also observed that MnO_2_ can act as a binder to combine the PEO chains, which confirms the assumption that the Li conductivity and mechanical performance are improved by the addition of MnO_2_ nanosheets [31]. These studies indicate that DFT can be fruitfully applied to the study of heterostructures for Li-ion battery applications. Additionally, the electronic transport properties, namely, electrical conductivity, (EC) can also be explored when different materials are interfaced, forming hybrid cathodes or anodes, to elucidate the effect of short-chain organic binders such as lysine introduced at the interface.

In this study, we started from flat TiO_2_ as a model substrate for titanate surfaces and follow our previously developed protocol where we formed uniform and thickness-controlled graphene coatings on TiO_2_ using PLL as an interfactant [32]. On that experimental basis and inspired by promising insights gained from first-principles calculations, we demonstrate the coating of mesoporous lithium titanium oxide microparticles [33] and reveal the performance of rGO/PLL/LTO and rGO/LTO with respect to their anode material rate capacity.

## 2. Experimental Section

Below, we describe in more detail the applied simulation and characterization approaches as well as the materials synthesis.

### 2.1. Computational Details

Using Hubbard-corrected density functional theory (GGA+U) as implemented in the Quantum ESPRESSO package [34] in the first stage, the structures of bulk Li_4_Ti_5_O_12_ (LTO), different LTO surfaces (bare and functionalized), and LTO/rGO heterostructures (LTO/rGO and LTO_/_Lysine/rGO) were relaxed. The exchange-correlation energy was represented by the Perdew–Burke–Ernzerhof functional (PBE) within the generalized gradient approximation (GGA) [35]. The valence and core electron interactions were described by pseudo-potentials from the PSlibrary database [36]. The Kohn–Sham orbitals were expanded using a plane-wave basis with a 40 Ry energy cutoff. The Fermi–Dirac smearing method was employed with the smearing parameter of 0.01 Ry [37]. Geometry optimizations were carried out with the convergence thresholds of 10^−3^ and 10^−5^ Ry for the total energy and the force on each atom, respectively, and the Li_4_Ti_5_O_12_ crystal structure was taken from databases of the Materials Project [38].

The direct-current conductivity tensor (DCCT) was calculated for the different surfaces (bare, functionalized, and heterostructures) using the Kubo–Greenwood Electrical Conductivity (KGEC) code [39]. The electrical conductivity (σ) appears in the differential form of Ohm’s law as $\vec{J}=\sigma \;\vec{E}$. In general, it is an anisotropic quantity, namely it may vary in different directions inside the material so that it physically is represented as a frequency-dependent complex tensor [39,40]. The direct current electric transport properties of an anisotropically structured material can be described by the Kubo–Greenwood relation as a multidimensional summation:

$$\sigma_{dc}=-\frac{2e^{2}\hbar^{3}}{m_{e}^{2}\Omega }\underset{k}{\sum }w_{k}[\frac{2}{\delta }\underset{n}{\sum }\frac{\partial f\left(\epsilon_{nk}\right)}{\partial \epsilon_{nk}}\;ℜ\left(\left\langle \Psi_{nk}\left|\;\nabla \;\right|\Psi_{nk}\rangle \;\langle \Psi_{nk}\left|\;\nabla \;\right|\Psi_{nk}\right\rangle \right)\;+\frac{2}{\delta }\underset{\frac{n\neq n^{\prime }}{\epsilon_{nk}=\epsilon_{n^{\prime }k}}}{\sum }ℜ\left(\left\langle \Psi_{nk}\left|\;\nabla \;\right|\Psi_{n^{\prime }k}\rangle \;\langle \Psi_{n^{\prime }k}\left|\;\nabla \;\right|\Psi_{nk}\right\rangle \right)\;\;\;\;\;\;\;\;-\frac{{\Delta f}_{\;n\;\prime k,nk}}{{\Delta f}_{nk,\;n\;\prime k}}ℜ\left(\left\langle \Psi_{nk}\left|\;\nabla \;\right|\Psi_{n^{\prime }k}\rangle \;\langle \Psi_{n^{\prime }k}\left|\;\nabla \;\right|\Psi_{nk}\right\rangle \right)\frac{\delta /2}{{\left(\Delta \epsilon_{nk,nk^{\prime }}\right)}^{2}+\delta^{2}/4}]$$

where Ω is the unit cell volume, *W***_k_** is the **k**-point integration weights and *f*($\epsilon_{nk}$) denotes the Fermi–Dirac distribution function.

### 2.2. Materials

Titania was sputter-deposited on silicon oxide substrates using a low-pressure plasma-enhanced chemical vapor deposition (PECVD) process. The home built low-pressure plasma chamber was equipped with a TRUMPF Hüttinger Quinto system for radio frequency generation (13.56 MHz). More detailed information about the coating process is specified elsewhere [41]. A commercial GO dispersion (4 mg/mL Graphenea) was purified before use as follows: 1 mL of the commercial GO solution was added to an Eppendorf pipette tip and centrifuged for 3 min (min). The supernatant was discharged, and the sedimented pellet was dispersed in 1 mL of ultrapure water using vortex agitation and ultrasound. The procedure was repeated five times, and the finally obtained pellet was dispersed in ultrapure water. The suspension was used to prepare formulations containing 25 µg/mL or 100 µg/mL GO in the MES based on 2-(N-morpholino)ethanesulfonic acid) buffer. MES buffer was prepared with ultrapure water and Sigma-Aldrich reagents. The used PLL in this work was poly-L-lysine hydrochloride with a molecular weight between 15 and 30 kDa (Sigma-Aldrich, Darmstadt, Germany). Anhydrous THF (containing 250 ppm butylated hydroxytoluene (BHT) as an inhibitor, 99.9% purity), titanium (IV) isopropoxide (97%), lithium ethoxide solution 1.0 M in THF, and oxalic acid (99.0% purity) were purchased from Sigma-Aldrich (Darmstadt, Germany). Polyvinylidene fluoride (Kynar R HSV 900 PVDF,) was supplied to us by ARKEMA Innovative Chemistry (Nanterra, France). Anhydrous 99.5% N-methyl-2-pyrrolidone (NMP) was purchased from Sigma-Aldrich. 1M lithium hexafluorophosphate in ethylene carbonate: dimethyl carbonate 1:1 (*v*/*v*) (1 M LiPF_6_ EC:DMC) as an electrolyte was bought from Solvionic (Toulusse, France).

#### 2.2.1. Synthesis of Li_4_Ti_5_O_12_ (LTO) Nanostructured Material

LTO nanostructured material was synthesized via a sol-gel reaction, the details of which can be found in a previous study [33]. Briefly, the glassware was initially placed in a vacuum oven over night at 100 °C to remove humidity. The reaction starting from the educts was carried out under a nitrogen (N_2_) atmosphere. An aliquot of 62 mL of tetrahydrofuran (THF) was injected into a round flask under stirring, then the educt of 1.81 mL lithium ethoxide was added before slowly adding 0.65 mL titanium (IV) isopropoxide. The solution was stirred for two hours. Prior to the synthesis, a 5.6 wt% stock solution of oxalic acid, the polystyrene-*b*-poly (ethylene oxide) (PS-b-PEO) block copolymer (BCP), and PS homopolymer (HP) in anhydrous THF were prepared. Then, 3.956 mL oxalic acid was added, and following that, 2.019 mL of a PS-b-PEO BCP (average molecular weight Mw: PS (60,000 g/mol)—PEO (14,500 g/mol), Polymer Source Inc, Quebec, Canada) and 10.1 mL of PS HP (average molecular weight of Mw (35,000 g/mol)) in THF were added. Then, the reaction temperature was ramped from 40 °C to 120 °C over the course of two days. Finally, the reaction mixture was placed in a furnace under an argon atmosphere with a flow of 4 L/min, and the temperature was increased to 800 °C for 4 h.

#### 2.2.2. TiO_2_ and LTO Functionalization

TiO_2_ substrates with an area around 1 cm^2^ were submerged in the PLL solution for 1 h and then rinsed with MES buffer and Milli-Q water three times. 100 µL of 0.25 µg/mL (sample A) suspension was pipetted on the LTO/PLL surface for 10 min or 100 µg/mL GO for 30 min (sample B), and then the substrate was gently rinsed with Milli-Q water. For LTO particles, 2 mg of LTO were dispersed in 2 mL of 0.1 mg/mL of PLL in 30 mM MES at pH = 6.1 and were left to react for one hour. The particles were precipitated at 5000 rpm using a microcentrifuge. Then, they were rinsed in Milli-Q water and redispersed. 2 mg of the dried LTO/PLL powder was redispersed in 2 mL of the 0.25 µg/mL GO dispersion for 10 min and then precipitated and rinsed three times with Milli-Q water.

#### 2.2.3. X-ray Diffraction Technique

A Rigaku Ultima IV (Applied Rigaku Technologies, Inc., Austin, TX, USA) with a copper target (K alfa) was used to carry out X-rays diffraction experiments.

#### 2.2.4. Scanning Electron Microscopy (SEM)

Scanning electron microscopy of the flat TiO_2_ functionalized samples was carried out on a FEI Helios 600 Dual-Beam machine (Austin, TX, USA) with a resolution of about 1 nm. We chose an accelerating voltage of 1kV and low currents to be as surface sensitive and protective of the samples as possible. We also did not apply a conductive layer onto the sample, because on one hand, the flakes should be conductive, and on the other hand, a good contrast difference between the sample surface and the flakes was obtained. Secondary-electron images were recorded either with an Everhardt–Thornley or through-lense detector, while backscattered images were recorded with a dedicated circular backscatter detector.

SEM images of the LTO microparticles were acquired on a Tescan Mira 3 LMH scanning electron microscope (Thermo Fisher Scientific, Waltham, MA, USA) at accelerating voltages of 18 kV.

#### 2.2.5. Thermogravimetric Analysis (TGA)

TGA was performed on a TGA/DSC 1 instrument (Mettler Toledo Greifensee, Switzerland) in a temperature range of 25 to 600 °C with a heating rate of 10 °C min^−1^ under an N2 flow of 30 mL min^−1^.

#### 2.2.6. Attenuated Total Reflectance (ATR-FTIR)

FTIR spectra were recorded on a ALPHA II series spectrometer (Bruker, Ettlingen, Germany) equipped with an ALPHA’s Platinum ATR single reflection diamond ATR module. Spectra were averaged over 32 scans using a resolution of 2 cm^−1^ over the IR range of 4000–400 cm^−1^. The data were analyzed using Bruker OPUS software (version 8.1).

#### 2.2.7. X-ray Photoelectron Spectroscopy (XPS) 

XPS spectra for compositional surface analysis were taken using a Kratos Axis Ultra system (Kratos Analytical Ltd., Manchester, UK) with a monochromatic Al Kα source operated at a base pressure of 4 × 10^−8^ Pa, low-energy electrons (<5 eV) for sample neutralization, and a 0° electron take-off angle with respect to the surface normal. For high-resolution spectra, 10 eV and 20 eV analyzer pass energies were used, whereas for survey spectra, a 160 eV analyzer pass energy was employed. The probe area was elliptical in shape, having major and minor axes of 700 μm and 300 μm, respectively. At these conditions, XPS probes the sample at approximately 10 nm of depth from the incident surface. The binding energy values were referenced by positioning the C1s signals for the pre-dominating hydrocarbonaceous species at 285 eV [28,41]. Surface concentration values were calculated based on the simplifying geometric model assumption that the sample surface is homogeneously composed. Thus, such concentration values contribute to facilitating a quantitative comparison between distinct surface states.

#### 2.2.8. Atomic Force Microscopy (AFM)

For structural surface characterization, AFM images of the TiO_2_ substrates were obtained using a scanning probe microscope (Asylum research, Santa Barbara, CA, USA) operated in tapping mode. Si-tipped cantilevers (tip radius ≈ 5 nm) having ≈ 250 kHz resonance frequencies (force constant k ≈ 0.7 N m^−1^) were used. The software Asylum User version 16.31.232 was applied to analyze the data. For the KPFM cantilevers with tips coated with a double layer of chromium and platinum iridium (Nanosensors, Neuchatel, Switzerland), a 75 kHz resonance frequency and a force constant around 2.8 N/m were used.

#### 2.2.9. Electrochemical Properties of Mesoporous LTO Microspheres

To characterize the electrochemical performance of the synthesized mesoporous LTO microparticles, composite electrodes were prepared with LTO/PLL/rGO microparticles or LTO/PLL/rGO with poly (vinylidene fluoride) (PVDF) at a ratio of 8:0.2 using aluminum foil as the current collector. The slurry was cast onto aluminum foil and subsequently doctor bladed into a 100-μm thick electrode film, and then dried under a fume hood for two days. The dried electrode film was cut into 7/16-inch diameter discs, vacuum dried overnight at 100 °C, and then transferred into an argon-filled glovebox for assembly into Swagelok cells. A 1/2-inch diameter lithium metal chip was used as the counter electrode and a grade-GF/B glass microfiber filter was used as separator. Galvanostatic charge–discharge tests were conducted using an Arbin BT 2043 multiple channel cell test system in a voltage range of 1.0 to 2.5 V (vs. Li+/Li).

## 3. Results

Our preliminary studies had suggested that thin and stable GO layers could be formed on TiO_2_ substrates applying PLL as an interfactant, preferentially at the pH values of the aqueous formulations close to the isoelectric point of PLL [32,42]. This study aims to evaluate the use of PLL as an environmentally friendly interface binder with control of the layer thickness between graphene-oxide- and titanium-oxide-based materials for energy applications.

**Figure 1:**
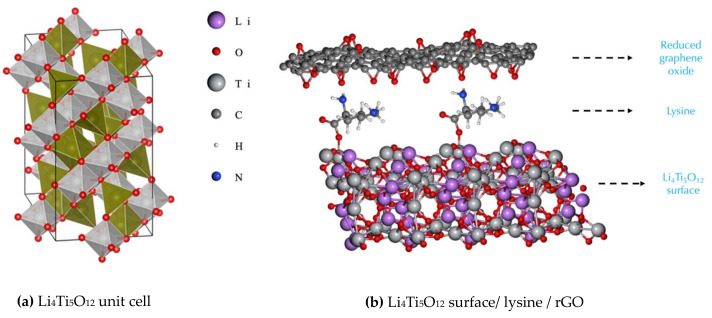
(**a**) Unit cell of Li_4_Ti_5_O_12_. Titanium atom cores occupy octahedral sites (grey), lithium atom cores occupy tetrahedral and octahedral sites (green), and oxygen atoms cores are shown in red. (**b**) A 2 × 1 × 1 supercell of Li_4_Ti_5_O_12_ surface/Lysine/rGO (cell boundaries are not shown for clarity).

To determine the effect of PLL on the electrical conductivity at the interface, a computational study was performed using Li_4_Ti_5_O_12_ since it is the most studied titanium-oxide-based material for battery fabrication. Li_4_Ti_5_O_12_ consists of a monoclinic (2/m) oxide ion lattice with lithium and titanium ions occupying either the tetrahedral or octahedral sites, showing a spinel structure, as seen in Figure 1a:

When designing composite electrodes, contact resistances are governed by the anchoring of molecules and electrical transport processes occurring at the interface between particulate condensed phases and their environment. With crystals being terminated by faceted surfaces, first-principles modelling of different surface orientations provides an insight into the likely stable surfaces of the crystal based on the calculated energies. First-principles modelling can also reveal which surfaces are more conductive, in order to find a good candidate to be used for modelling the whole LTO surface/lysine/rGO hybrid anode system, as seen in Figure 1b. Therefore, we carried out first-principles calculations to answer two main questions: What is the most likely surface of LTO, and how is the electrical conductivity of the LTO surface affected by the anchoring of lysine molecules? To address these questions, first, a set of six surfaces of Li_4_T_i5_O_12_ were created, and their electrical conductivity and energy-per-atom values were calculated for bare and lysine-functionalized terminations, with the results presented in Table 1 and Table 2, respectively.

Since the (111) surface showed the lowest energy, two orientations of the lysine molecule over the surface were explored, namely: (111)^α^ and (111)^β^. It is important to mention that different orientations for the anchoring of lysine on the (111) surface (alpha and beta) were explored in order to find the most stable (lowest energy) lysine configuration. Since the (111)^β^ surface was found to be significantly lower in energy and higher in EC than the other candidate surfaces, no further molecular orientations were explored on the other surfaces.

According to our DFT calculation results shown in Table 2, the (111)^β^ surface presents the highest EC value and the lowest energy value per atom. Therefore, in the absence of kinetic energy barriers for its formation, the (111)^β^ surface is the most likely functionalized Li_4_Ti_5_O_12_ crystal surface to exist. Due to these two features (highest electrical conductivity and lowest energy per atom), the (111) surface (with both of its configurations) was chosen as the surface to model the LTO _(111)_/Lysine/rGO hybrid anode material.

### Conductivity Modelling: LTO/Lysine/rGO

**Figure 2:**
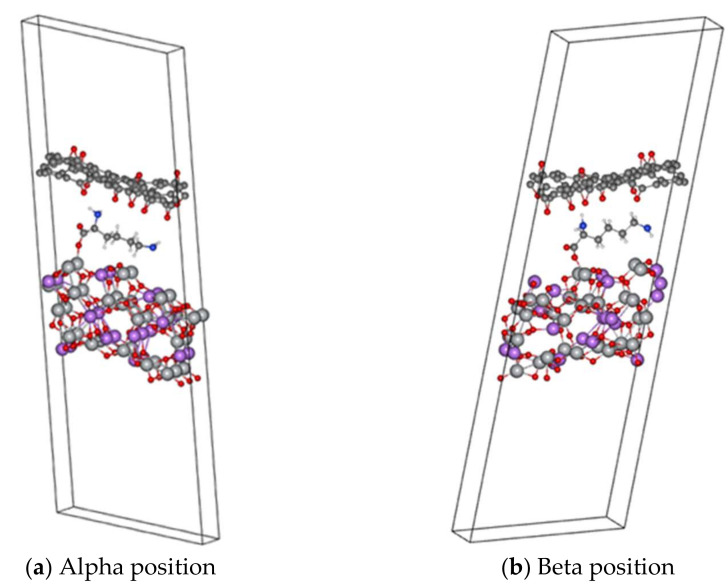
Slab representation for anchoring the lysine molecules on the (111) Li_4_Ti_5_O_12_, shown at the bottom, while at the top, a partially epoxidized rGO surface is depicted. (**a**) Lysine molecule attached on α position. (**b**) Lysine molecule attached on β position.

To model and study the EC values for the anode configuration, two approaches were used: first, modelling it as a slab (periodic along the *xy* direction in Figure 2), where two ways of orienting lysine over the (111) surface were explored, those being alpha (Figure 2a) and beta (Figure 2b). Our results suggest that the lysine molecule is bonded to the LTO surface by a covalent bond formed by one oxygen atom (belonging to the carboxyl group) and one Ti atom from the LTO, and the other interaction present that keeps lysine bound to the rGO is the hydrogen bonding, formed by the H-atoms (from nitrogen) and the O-atoms of the rGO.

**Figure 3:**
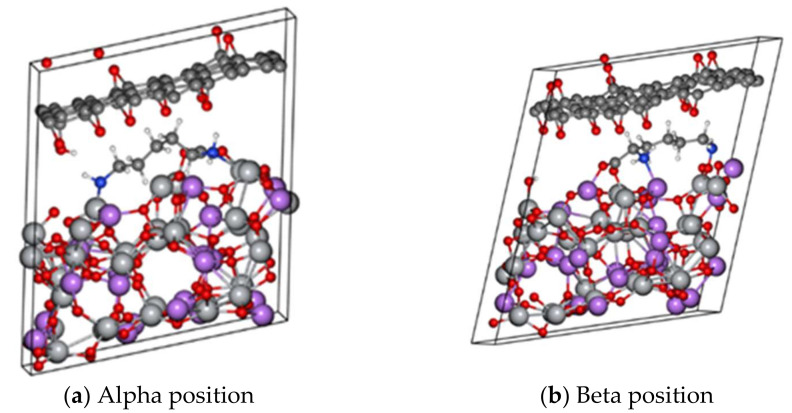
Anchoring of the lysine molecules on the (111) Li_4_Ti_5_O_12_ surface as bulk. (**a**) Lysine molecule attached on α position. (**b**) Lysine molecule attached on β position.

Second, the same previous approach was used, but the cell was modelled as a superlattice (periodic along the xyz direction in Figure 3), that is, with no vacuum along the z-axis.

Since the EC is an anisotropic quantity, it varies in different directions inside the material, so that is generally represented as a tensor:

$$\sigma =\left(\begin{array}{ccc} \sigma_{xx} & \sigma_{xy} & \sigma_{xz}\\ \sigma_{yx} & \sigma_{xy} & \sigma_{xz}\\ \sigma_{zx} & \sigma_{xy} & \sigma_{xz} \end{array}\right)$$

**Figure 4:**
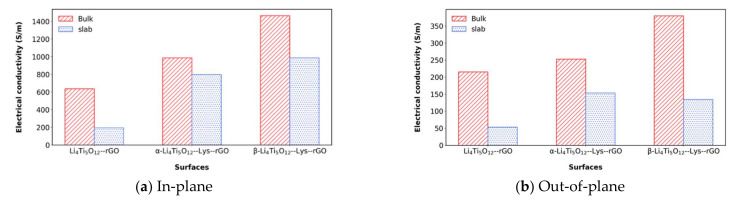
Comparison of EC values exploring two approaches (bulk and slab) along two orientations: (**a**) in-plane, and (**b**) out-of-plane.

Therefore, Table 3 shows the effect of lysine on the electrical conductivity when it is used as a binder between LTO and rGO. The obtained EC values are also summarized graphically in Figure 4. First, for the open system (slab), the introduction of lysine between rGO and LTO increases the electrical conductivity from 193.7 S/m for the LTO/rGO system up to 987.3 S/m for the β-LTO/lys/rGO. The EC values for β-LTO/lys/rGO are also higher than those of the bare (111) LTO surface. The change in the EC from LTO/rGO to β-LTO/lys/rGO is quite large, corresponding to an increment by a factor of approximately five. A slightly smaller increase is obtained in case of the α-configuration in α-LTO/lys/rGO. Similarly, for the superlattice configuration, the introduction of lysine in the α position leads to an EC increment of 155%, while the introduction of lysine in the β position leads to an EC increment of 230% relative to the EC of the superlattice configuration with no lysine between rGO and LTO. As expected, the electrical conductivity presents a higher value in the superlattice systems, since there are more atoms per unit volume, that is, there are more atoms inside the unit cell that contribute to the electronic transport. 

The obtained results for the slab and bulk systems, with similar trends for both the in-plane and out-of-plane configurations, indicate two conclusions: The presence of lysine as a binder clearly increases the electrical conductivity, favoring the electrical transport between rGO and Li_4_Ti_5_O_12_, and the most likely Li_4_Ti_5_O_12_/lys surface to exist is the (111)^β^ surface. In addition, these results pave the way for a more detailed computational analysis, showing which surface to explore and where to anchor the binder molecule [43].

The target was to find the potential effects of introducing an allegedly rather poorly electronically conductive lysine layer between neighboring LTO and rGO surfaces. We found indications for an enhancement of the conductivity relative to LTO and rGO and for a quantitatively considerable impact. Based on these findings, we expect that by increasing the coverage (in-plane) of lysine molecules that are present between the Li_4_Ti_5_O_12_ surface and the rGO surface, the electrical conductivity might be further increased. Thus, our results suggest that lysine provides enhanced conduction pathways between rGO and LTO, which substantiates its suggested use as an interfacial binder. In these molecular contacts, the Fermi level of the metal usually lies between the highest occupied molecular orbital (HOMO) and lowest unoccupied molecular orbital (LUMO) electronic states of the molecule. A small applied voltage results in electron transport through the molecule via nonresonant tunneling or, for higher voltages, via resonant tunneling through a molecular energy level [44,45]. The charge transport ($T_{mol}$) through the molecule is quantified by a ‘‘*β*’’ parameter (controlled by the molecular orbital energies and wave-functions) [46], where the tunneling probability is proportional to $e^{-\beta L}$, and L is the length of the molecule [46], and therefore the current is found to decrease exponentially with the increasing molecule chain length [46,47,48,49,50].

(1) $$T_{mol}=e^{-\beta \cdot L}$$

As a further step, we also explored the effect of a second lysine molecule along the vertical direction (out-of-plane), and as we stacked a second lysine molecule within the system, we observed a decrease in EC values, since the L parameter in Equation (1) became larger. This reveals that by increasing the number of lysine vertically (out-of-plane), this causes a detriment in the ease of electrical conduction, showing that for good performance, no more than one molecule is required (along the out-of-plane direction).

**Figure 5:**
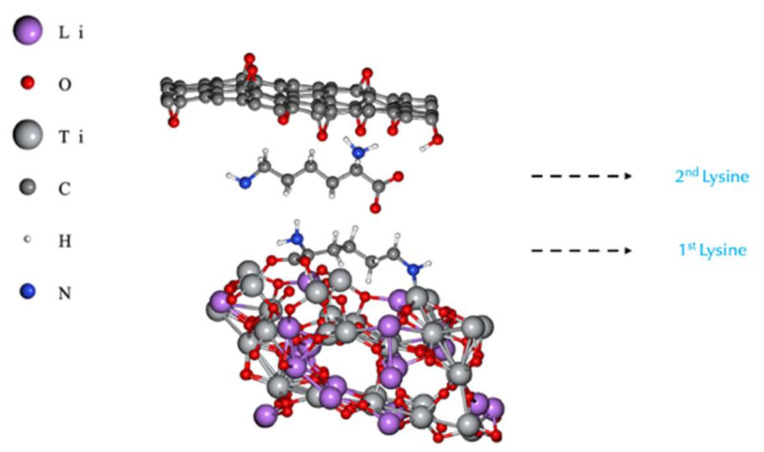
Stacking of two lysine molecules along the out-of-plane direction for the slab approach (cell boundaries are not shown for clarity).

By comparing the EC values of LTO/lys/rGO in Table 3 (in which there is only one lysine molecule) for the slab against the configuration shown in Figure 5 (with two lysine molecules), the EC value for in-plane decreased from 987.3 S/m to 251.9 S/m, and for out-of-plane decreased from 134.6 S/m to 66.8 S/m. These results indicate concordance between Equation (1) and the data obtained by DFT. In this case, the presence of a second lysine molecule changes how the first lysine interacts with the LTO surface, giving rise to an interaction of nitrogen with the surface of the LTO, and the second lysine is held together just by hydrogen-bond-type interactions.

Encouraged by the positive previous results obtained from the DFT simulations that showed an increment in the EC value and chemical stability by using one lysine molecule as a binder between the rGO and the LTO, we examined experimentally the performance of adsorbed PLL thin layers and the use of the polymer as a binder experimentally.

Before applying the previously developed methodology for batch processes with LTO particles, we investigated by XPS the formation of rGO in adsorbate systems formed from PLL/GO layers on a flat and smooth TiO_2_ model substrate (root mean square roughness 1.2 ± 0.3 nm) by applying an appropriate thermal treatment. While GO contains phenolic hydroxyl and carboxyl groups on its surface and basal-plane hydroxyl and epoxy groups [51], the partially reduced rGO shows a significantly higher electrical conductivity that can be obtained by applying temperatures higher than 200 °C [52]. We followed the layer-by-layer assembly protocol developed by Pinheiro et al. to form first a thin PLL layer at pH 6.1 and then a thin GO layer at pH = 6 [32]. Table 4 shows the atomic surface concentration of the respective samples before and after annealing at 227 °C in air. Two distinct TiO_2_/PLL/GO sample specimens are reported, with specimen B having been obtained from a dispersion with a higher GO concentration as compared to specimen A. The GO coverage was found to be three times higher for specimen B, and from a GO layer thickness of 3 nm, we infer that more than a GO multilayer had become deposited. This indicates that for preparing well-defined GO adsorbates on TiO_2_/PLL, the deposition process parameters need to be tailored thoroughly. 

**Figure 6:**
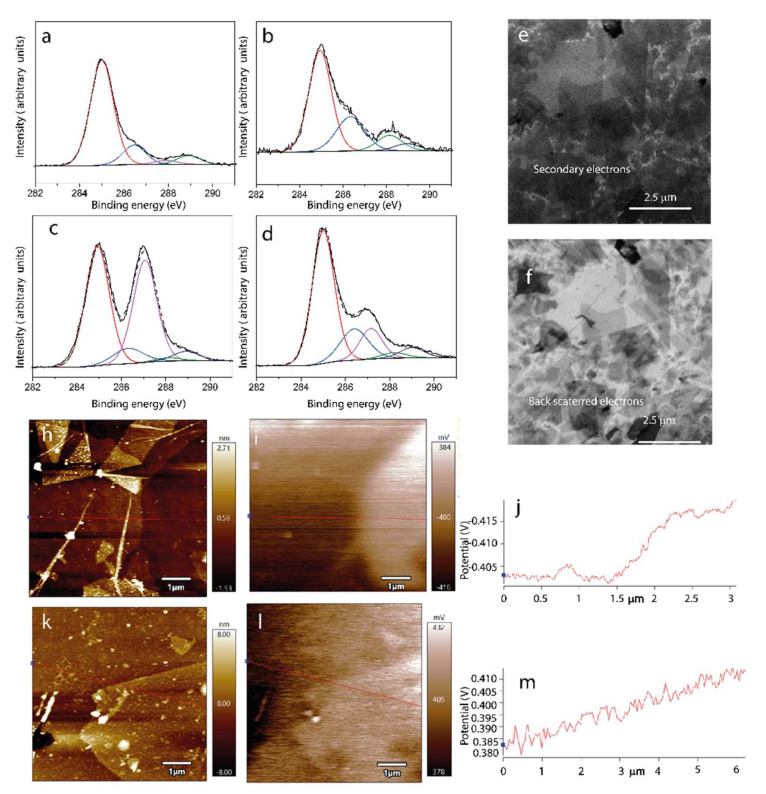
High resolution C1s XPS spectra of (**a**) TiO_2_, (**b**) TiO_2_/PLL, (**c**) TiO_2_/PLL/GO (sample B), and (**d**) TiO_2_/PLL/rGO (samples B). Scanning Electron Microscopy (SEM) images recorded using detection of (**e**) secondary electrons (SE-SEM) and (**f**) backscattered electrons (BSE-SEM) for a region of TiO_2_/rGO/PLL. AFM topography images for (**h**) TiO_2_/rGO and (**k**) TiO_2_/PLL/rGO. Images (**i**) and (**l**) show KPFM surface potential images for the regions shown in (**h**) and (**k**), respectively. Images (**j**) and (**m**) show the cross-section profiles along the lines depicted in (**i**) and (**l**), respectively.

The elements detected in higher concentrations composing the sample surfaces were related to titanium, oxygen, carbon, and nitrogen species. The layer thickness was calculated according to the protocol described by Corrales et al. [42], based on the attenuation of the Ti2p substrate signal intensity. PLL adsorption on the surface is expected to increase the concentration of hydrocarbonaceous and nitrogen-containing species, and the surface concentration [N] acts as a specific marker for the coverage with the polyamino acid. When comparing TiO_2_/PLL/GO (sample A) and TiO_2_/PLL/GO (sample B) in both states, “as-deposited” and “annealed”, the respectively obtained adsorbate layer thickness indicates a GO coverage around one monolayer for sample A and between two and three monolayers for sample B. The chemical moieties characteristically contributing to the C1s signals of PLL and GO, respectively, are hydrocarbonaceous species C*–C and C*–H (C1s binding energy B.E. around 285.0 eV), C*–N and C*–OH (effectively making for a signal at B.E. 286.4 eV [27]), epoxy-type C*–O–C (B.E. 287.1 eV [28]), amide/peptide C*O–N and carbonylic C*=O (B.E. around 288.2 eV), and carboxylic C*O–O (B.E. 289 eV [28]). The difference of the C1s signal’s shape of the TiO_2_/PLL/GO (sample B) in the as-deposited and annealed states, respectively, shows the partial reduction of GO that is adsorbed on the PLL-modified titania substrate, as seen in Figure 6a–d. The decrease in the oxygen surface concentration [O] while signals related to the titania substrate increase suggests a partial reduction in the oxygen-containing species due to the formation of rGO upon annealing. As with the [Ti], the [N] also increased, and evidence for a decomposition of the polylysine was not found. The annealing at 227 °C clearly results in a reduction in the epoxy-related peak and an intensity increase for C*–OH, which is attributed to the epoxy ring opening. The reduction in GO thus results in the formation of (partially) reduced GO and is concomitant with a minor restructuring of the respective layer since the layer thickness of the annealed specimen was found to be decreased by 10%.

SEM images of the TiO_2_ surface after the adsorption and thermal treatment of PLL-rGO adsorbates show a high-density coverage of laterally overlapping flakes. While the SE-SEM image in Figure 6e reveals a few aggregates as dark features, the BSE-SEM image in Figure 4f shows a different contrast between the aggregated flakes and flake regions in the second or first rGO layers, indicated by a more-or-less decreased brightness as compared to TiO_2_ substrate regions that are not covered by flakes. In Figure 6e,f we could observe only in the flat surfaces functionalized with the rGO, PLL/rGO/TiO_2_ samples, with isolated islands that appear dark and percolating network flakes that appear bright, especially when connected to the electrically grounded region of the sample that is displayed in the bottom part of the image [53]. The regions with a darker contrast are related to regions in contact with the grounded and the comparatively weakly conductive TiO_2_ substrate. As shown in Supplementary Figure S1A, an introduced scratch highlights the contrast change produced between the flakes interconnected in the percolating network and the material that is laterally disconnected from the interrupted network. We attribute the observed differences to two effects: on the one hand, the material contrast between titanium-rich and carbon-rich regions, and on the other hand, regionally differential surface charging due to distinctly conductive percolation paths through laterally overlapping flakes that are vertically separated by polymer molecules. The only minor charging of the flakes behind the scratch (displayed at the top of the image in Supplementary Figure S1B) suggests that the flakes there still have a sufficient interaction with the surface in a way that the electrons can move through the vertically layered network in an out-of-plane direction. The rGO–rGO unions appear to produce charging in the network.

Extrapolating from the electron conduction paths in the laterally extended and grounded flat-layered TiO_2_/PLL/rGO system, we may expect similar percolation paths to be effective in 3D bulk geometries of composites with laterally bordered particulate fillers. In this case, the percolation properties can additionally be affected when the filler forms aggregated structures that are then interconnected by individual filler particles. Often, such agglomeration can promote percolation. In a similar manner, the fillers can form a phase-separated, co-continuous morphology consisting of graphene-rich and poor phases within the volume of the composite. This is known as selective localization [53], and often leads to a conductive composite at lower loadings.

Surface potential mappings were acquired to determine the PLL adsorption following the changes in the work function of single flat rGO flakes on the TiO_2_ or on the TiO_2_/PLL [54,55]. Voltage signal changes from—to + from TiO_2_/rGO to TiO_2_/PLL/rGO were reproducibly observed for flake-covered substrate regions and thus are attributed to changes in the surface dipole direction. Other studies also indicated that the functionalization of PLL layers on gold or stainless surfaces led to a shift from negative to positive surface potentials [56]. Figure 6k shows the topography of single rGO flakes on the surfaces under study, and it is observed that flakes in rGO single layers on PLL have an average height of (1.3 ± 0.2) nm above the underlying PLL-coated substrate.

The surface potential differences (SPD) were calculated based on the KPFM findings obtained from 17 line scans for 5 different flakes as compared to the underlying and circumjacent substrate, as seen in Figure 6h–m. The average calculated differences are (15 ± 3) and (11.0 ± 2.3) mV, respectively. The results suggest that the adsorbate modified the TiO_2_ surface and/or the rGO. Amines are reported to decrease the work function of graphene, having an n-type doping effect on graphene [57]. Changes in the 10-mV range have been reported for biomolecules adsorbed on different surfaces and forming thin layers [58]. The carrier conduction process depends on the work function differences of LTO and graphene and LUMO and HOMO of the PLL thin film [59], suggesting a conductivity change in the system.

Finally, we present the characterization outcomes achieved after preparing LTO particles encased by rGO.

**Figure 7:**
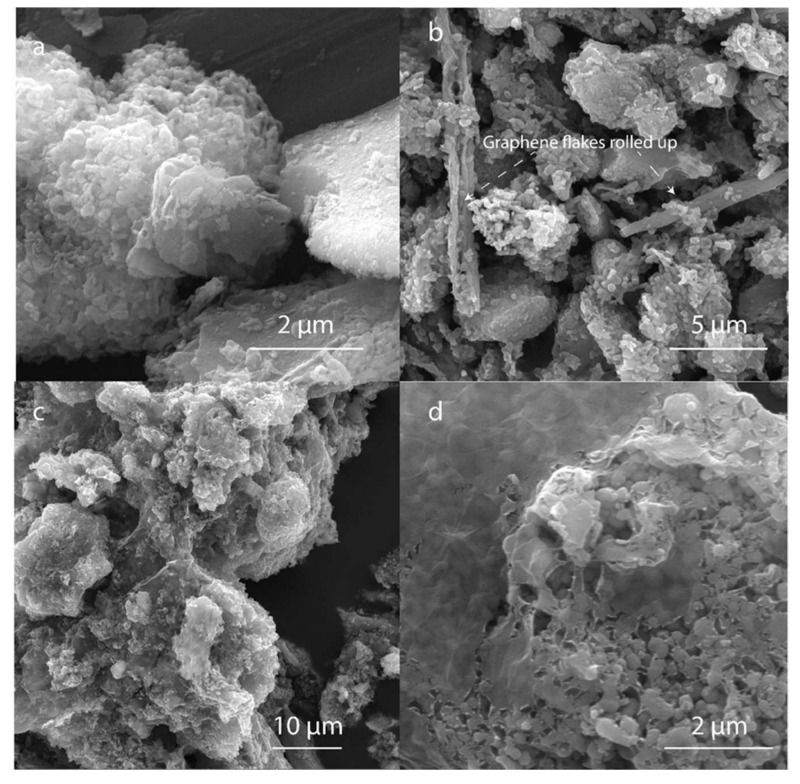
Scanning Electron Microscopy (SEM) secondary electrons (SE-SEM) of (**a**) LTO powder, (**b**) LTO/rGO, (**c**) and (**d**) LTO/PLL/rGO.

Figure 7a shows the LTO microparticles control, which are approximately (5.0 ± 1.3) µm in length. Figure 7b shows the LTO coated with the rGO without the PLL or other binders after GO reduction. The rGO tended to roll up and did not attach to the LTO. On the other hand, the particles coated with PLL/rGO tend to form particle aggregates, forming a percolation cluster. Thin layers with few amounts of micrometric wrinkles of rGO are formed on top of the particles and contribute to the particle–particle clustering. The microscopic findings indicate that a layer-wise assembly was implemented and that similar findings for their topography and thickness are expected for the layers formed on LTO as for the layers formed on TiO_2_ when using the PLL approach described in previous sections. Thin-layer and well-dispersed rGO is desirable to decrease the amount of carbon that does not contribute to the contact area with the LTO surface, because it is the constituent that helps to enhance the electrical conductivity but decreases ionic conductivity and also increases the volume and weight of the electrode [60]. However, obtaining a completely wrapped particle without proving ion conduction paths might not allow for sufficient ion transport and therefore would be unfavorable [61]. The rGO was observed to present few wrinkles. Amad et al. showed that the electrical conductivity of exfoliated graphene presented conductivity differences on step edges and wrinkles in comparison to flat regions. They suggested that the pattern of the atoms on the wrinkles lowers the local conductance due to modification of the band structure [62].

**Figure 8:**
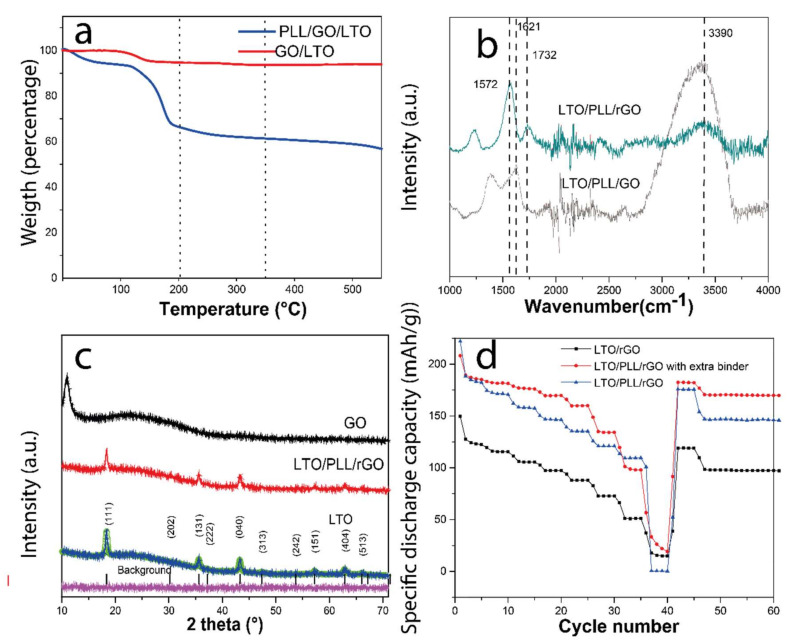
(**a**) Thermogram of LTO/GO and LTO/PLL/GO powders. (**b**) ATR-FTIR spectra of the LTO/PLL/GO before and after reduction at 227°. (**c**) XRD spectra of GO, LTO/PLL/rGO, and LTO. The green line shows the fitted Rietveld refinements data of the LTO. The black vertical bars indicate the peak positions for spinel LTO. The peaks for a spinel structure (space group: Fd-3m) are indexed with the (hkl) values. (**d**) Discharge rate capacity tests of LTO/rGO and LTO/PLL/rGO of composite electrodes. Cycle testing at a C-rate of 10.

Following thermogravimetry, the content of PLL adsorbed on LTO was (1.4 ± 0.9) wt%, and the rGO content was approximately (4.3 ± 0.2) wt% (calculated based on the GO and PLL reagents thermograms), as seen in Figure 8a. In contrast, the rGO deposited on LTO without the PLL at the interface amounted to only 1.21 wt%. These findings indicate a very significant improvement in GO adsorption due to the PLL modification of the LTO particles. For the sample control by electrical characterization, in view of the different rGO weight proportions adhered to the LTO, the rGO mass was adjusted to the same amount of carbon for LTO/PLL/rGO and LTO/rGO due to the changes in the conductivity depending on the rGO concentration. The samples underwent a thermal treatment to reduce the GO at 227 °C before testing their capacity as an anode material. In Figure 8b the FTIR spectra of the LTO/PLL/GO and LTO/PLL/rGO are displayed. Comparing them indicates that the amine peak at 3390 cm^−1^ decreased considerably and the peak at 1621 cm^−1^, corresponding to carboxylic groups and or amide groups, may be overlapped with the peak at 1572 cm^−1^ of C=C aromatic groups [63]. These results suggest that the PLL remained partially at the interface after the rGO reduction, as the PLL degradation temperature range is between 200 °C and 550 °C [63].

The XRD pattern of LTO in Figure 8c shows that the peaks are well indexed to those of a spinel Li_4_Ti_5_O_12_ structure (Fd-3m, COD card No. 96-100-1099), and no detectable impurity phases such as rutile or anatase are observed. The average crystallite sizes of LTO are 23.5 nm, as calculated using the XRD pattern and the Scherrer equation (K = 0.9) by averaging the values obtained from the (111), (131), (040), (151), and (404) planes. The peak corresponding to the GO presented at 10–13° is not observed in the TiO_2_/PLL/rGO, confirming the reduction of the rGO [64].

Finally, Figure 8d shows the rate capacity of charge and discharge (in order of the following C-rates: 0.1, 0.2, 0.5, 1, 2, 5, 10, 20; 5 cycles at each C-rate and back to 0.1C; 5 cycles, and 1C; 15 cycles) of the LTO anode material. The delivered discharge capacities of LTO anode half cells are clearly visible and show a higher capacity than the theoretical capacity of 175 mAh g^−1^ for one cycle [65], as expected due to the addition of the rGO. The LTO/PLL/rGO presented a much higher capacity than the LTO/rGO at all rates except at a C-rate of 20. These results suggest that the conductivity is enhanced due to the better adhesion of the rGO on the LTO surface. More importantly, the sample capacities mostly recovered to the initial values of about 182 and 119 mAh g^−1^ upon returning to the C-rate of 0.1 for LTO/PLL/rGO and LTO/rGO, respectively, and to about 170 and 97 mAh g^−1^ when returning to the C-rate of 1 for LTO/PLL/rGO and LTO/rGO, respectively. We observed that the PLL enhanced the particle aggregation to form a more compact structure for well-dispersed rGO, but this powder turned out not to be stable on the aluminum surface when it was casted. Therefore, 0.2 wt% with respect to the LTO mass of PVDF in NMP was added on top of the particulate film formed on the current collector to stabilize the powder. Figure 8d shows the improved rate capacity due to the enhanced contact with the aluminum foil. Further studies are necessary to determine other biodegradable binders and mixtures that could be used with the main purpose of stabilizing the powder in the aluminum current collector and to elucidate how these layers affect the solid electrolyte interface, the cracks, and dendritic formation.

## 4. Conclusions

DFT calculations suggested that the (111) surface is the most likely stable surface at the rGO–LTO interface. Furthermore, it shows a higher electrical conductivity compared to the other surfaces studied (000, 100, 010, 001 and 101). The comparison of the LTO/rGO and LTO/Lys/rGO systems used a hybrid anode as its model, which showed that the introduction of lysine at the interface leads to a significant increase in electrical conductivity (409%) of the rGO–lysine–LTO system relative to the rGO–LTO system, and of just 30% when two lysine molecules were stacked out of plane.

Experimentally, it was found that the capacity of the LTO/rGO electrode was improved by using thin layers of PLL as the binder. The interfactant layer improved the electron mobility and allowed for control of the layer thickness and assembly of the rGO layers. Understanding the electronic conduction at interfaces between biomolecules, 2D materials, and inorganic surfaces is the foundation for the development of next-generation biotechnology. Biomolecules are promising molecular junctions for tuning interface properties and forming innovative materials. In the future, this understanding can be used as a platform for applications such as batteries, but also for electrically conductive and mechanoresponsive sensors for interfaces in coatings or adhesive joints. Furthermore, it is of great interest to a wide range of fields and various applications ranging from bio-sensing, bio-catalysis, and biomedical research to nanoscale electronics, envisioning future bio-circuits and implantable electronics.

## Figures and Tables

**Table 1:** Electrical conductivity and energy per atom for six bare surfaces of Li_4_Ti_5_O_12._

Li_4_Ti_5_O_12_ Surface	Electrical Conductivity (S/m)	Energy per Atom (kcal/atom)
(000)	32.984	7376.343
(100)	49.591	838.227
(010)	78.365	844.188
(001)	639.987	0.062
(101)	47.258	658.885
(111)	656.584	0.0000

Energy per atom given relative to the lowest-energy surface.

**Table 2:** Electrical conductivity and energy per atom for six **functionalized surfaces** of Li_4_Ti_5_O_12_.

Li_4_Ti_5_O_12_ Surface	Electrical Conductivity (S/m)	Energy per Atom (kcal/atom)
(000)	30.045	7485.435
(100)	139.888	1826.711
(010)	1002.756	1830.539
(001)	510.639	128.796
(101)	66.756	7003.100
(111)^α^	282.465	126.129
(111)^β^	1794.006	0.0000

The energy values per atom were normalized with respect to the (111)^β^ surface.

**Table 3:** Electrical conductivity values (S/m) for the whole anode material, using the two adsorbate configurations (α and β), in-plane and out-of-plane (along the σ_zz_ direction).

Interphase	Slab (In-Plane)	Bulk (In-Plane)	Slab (Out-of Plane)	Bulk (Out-of-Plane)
Li_4_Ti_5_O_12_/rGO (no lysine in between)	193.7	636.0	52.6	215.4
α-Li_4_Ti_5_O_12_/Lys/rGO	798.3	985.8	153.8	253.1
β-Li_4_Ti_5_O_12_/Lys/rGO	987.3	1464.9	134.6	380.3
β-Li_4_Ti_5_O_12_/Lys/Lys-rGO	251.9		66.8	

**Table 4:** Atomic surface concentrations (in atomic %, at%) as obtained by XPS for flat TiO_2_ coated with PLL and GO in a layer-by-layer approach. Values are reported for as-deposited (labelled “as-dep.”) or annealed (labelled “annl.”) adsorbates and were obtained as average values from three regions each of different samples.

Samples	[O]		[Ti]		[N]		[C]		Layer Thickness	
	(at%)		(at%)		(at%)		(at%)		(nm)	
	as-dep.	annl.	as-dep.	annl.	as-dep.	annl.	as-dep.	annl.	as-dep.	annl.
									A	
Pristine TiO_2_	47.8	53.1	21.2	23.0	0.4	0.5	28.6	21.1		
TiO_2_/PLL	49.0	52.4	21.6	22.2	2.6	2.1	24.9	21.2	0.3	0.2
TiO_2_/PLL/GO, sample A	37.6	31.4	12.8	9.1	1.6	1.4	44.7	56.7	1.3	1.4
TiO_2_/PLL/GO, sample B	29.9	25.8	4.1	6.1	1.1	1.2	64	65.7	3.5	3.1

## Data Availability

Not applicable.

## Supplementary Materials

The following supporting information can be downloaded at: https://www.mdpi.com/article/10.3390/polym14112150/s1, Figure S1: Scanning Electron Microscopy (SEM) images recorded using detection of secondary electrons (SE-SEM) for TiO_2_/PLL/rGO A) and B) with and without introduced scratches, respectively.

## References

[1] Clark S., Bleken F., Stier S., Flores E., Welzel C., Andersen C.W., Marcinek M., Szczesna-Chrzan A., Gaberscek M., Palacin R. (2021). Toward a Unified Description of Battery Data. Adv. Energy Mater..

[2] Greaves M., Barg S., Bissett M. (2019). MXene-Based Anodes for Metal-Ion Batteries. Batter. Supercaps.

[3] Zhang F., Meng T., Gao A., Shu D., Chen H., Cheng H., Zhou X. (2019). In Situ Supramolecular self-assembly assisted synthesis of Li4Ti5O12−carbon-reduced graphene oxide microspheres for Lithium-Ion batteries. ACS Sustain. Chem. Eng..

[4] Wei G., Zhang J., Usuelli M., Zhang X., Liu B., Mezzenga R. (2021). Biomass vs inorganic and plastic-based aerogels: Structural design, functional tailoring, resource-efficient applications and sustainability analysis. Prog. Mater. Sci..

[5] Thackeray M.M., Amine K. (2021). Li4Ti5O12 spinel anodes. Nat. Energy.

[6] Chiou Y.-C., Olukan T.A., AlMahri M.A., Apostoleris H., Chiu C.H., Lai C.-Y., Lu C., Santos S., Almansouri I., Chiesa M. (2018). Direct Measurement of the Magnitude of the van der Waals Interaction of Single and Multilayer Graphene. Langmuir.

[7] Fu X., Zhong W. (2019). Biomaterials for high-energy lithium-based batteries: Strategies, challenges, and perspectives. Adv. Energy Mater..

[8] Divakaran A.M., Minakshi M., Bahri P.A., Paul S., Kumari P., Manjunatha K.N. (2021). Rational design on materials for developing next generation lithium-ion secondary battery. Prog. Solid State Chem..

[9] Mohsen M., Bahadorikhalili S., Lari N., Hadi M., Babaiee M., Eqra R. (2020). The Effect of Crystalline microstructure of PVDF binder on mechanical and electrochemical performance of Lithium-Ion Batteries Cathode. Z. Für Phys. Chemie.

[10] Kraytsberg A., Ein-Eli Y. (2016). Conveying Advanced Li-ion Battery Materials into Practice The Impact of Electrode Slurry Preparation Skills. Adv. Energy Mater..

[11] Richardson J.J., Cui J., Bjornmalm M., Braunger J.A., Ejima H., Caruso F. (2016). Innovation in Layer-by-Layer Assembly. Chem. Rev..

[12] Georgakilas V., Tiwari J.N., Kemp K.C., Perman J.A., Bourlinos A.B., Kim K.S., Zboril R. (2016). Noncovalent functionalization of gra-phene and graphene oxide for energy materials, biosensing, catalytic, and biomedical applications. Chem. Rev..

[13] Bostick C.D., Mukhopadhyay S., Pecht I., Sheves M., Cahen D., Lederman D. (2018). Protein bioelectronics: A review of what we do and do not know. Rep. Prog. Phys..

[14] Brancolini G., Bellucci L., Maschio M.C., Di Felice R., Corni S. (2019). The interaction of peptides and proteins with nanostructures surfaces: A challenge for nanoscience. Curr. Opin. Colloid Interface Sci..

[15] Qiu X., Chiechi R.C. (2022). Printable logic circuits comprising self-assembled protein complexes. Nat. Commun..

[16] Tanaka M. (2013). Physics of interactions at biological and biomaterial interfaces. Curr. Opin. Colloid Interface Sci..

[17] Basu S., Pacelli S., Paul A. (2020). Self-healing DNA-based injectable hydrogels with reversible covalent linkages for controlled drug delivery. Acta Biomater..

[18] Mehrotra P. (2016). Biosensors and their applications—A review. J. Oral Biol. Craniofacial Res..

[19] Stamboroski S., Joshi A., Noeske P.L.M., Köppen S., Brüggemann D. (2021). Principles of Fibrinogen Fiber Assembly In Vitro. Macromolecular Bioscience..

[20] Stamboroski S., Boateng K., Lierath J., Kowalik T., Thiel K., Köppen S., Noeske P.-L.M., Brüggemann D. (2021). Influence of Divalent Metal Ions on the Precipitation of the Plasma Protein Fibrinogen. Biomacromolecules.

[21] Dale-Evans A.R., Robinson M., Lloyd H., Gavaghan D., Bond A., Parkin A.A. (2021). Voltammetric Perspective of Mul-ti-Electron and Proton Transfer in Protein Redox Chemistry: Insights from Computational Analysis of Escherichia coli HypD Fourier Transformed Alternating Current Voltammetry. Front. Chem..

[22] Toigo C., Arbizzani C., Pettinger K.-H., Biso M. (2020). Study on Different Water-Based Binders for Li4Ti5O12 Electrodes. Molecules.

[23] Liebel C. (2020). Sustainable Battery Materials from Biomass. ChemSusChem.

[24] Ramkumar R., Minakshi M. (2015). Fabrication of ultrathin CoMoO4 nanosheets modified with chitosan and their improved performance in energy storage device. Dalton Trans..

[25] Bargnesi L., Gigli F., Albanelli N., Toigo C., Arbizzani C. (2022). Crosslinked Chitosan Binder for Sustainable Aqueous Bat-teries. Nanomaterials..

[26] Stamboroski S., Stachera P.N., Ureña Y.R.C., Hrycyna G.H., Neto W.I.T.R., De Azambuja W.K., Salz D., Ihde J., Noeske P.-L.M., Cavalcanti W.L. (2016). Implementation of diverse non-centrosymmetric layer concepts for tuning the interface activity of a magnesium alloy. Appl. Adhes. Sci..

[27] Ureña Y.R.C., Cavalcanti W.L., Soltau M., Villalobos K., Rischka K., Noeske P.-L.M., Brune K., Dieckhoff S. (2017). Interfactant action of an amphiphilic polymer upon directing graphene oxide layer formation on sapphire substrates. Appl. Adhes. Sci..

[28] Perez F.M., Ureña Y.R.C., Rischka K., Cavalcanti W.L., Noeske P.-L.M., Safari A.A., Wei G., Ciacchi L.C. (2019). Bio-interfactants as double-sided tapes for graphene oxide. Nanoscale.

[29] Pignanelli F., Romero M., Faccio R., Fernandez L., Mombrú A. (2018). Enhancement of Lithium-Ion Transport in Poly(acrylonitrile) with Hydrogen Titanate Nanotube Fillers as Solid Polymer Electrolytes for Lithium-Ion Battery Appli-cations. J. Phys. Chem. C.

[30] Deng Z., Mo Y., Ong S.P. (2016). Computational studies of solid-state alkali conduction in rechargeable alkali-ion batteries. NPG Asia Mater..

[31] Li Y., Sun Z., Liu D., Gao Y., Wang Y., Bu H., Li M., Zhang Y., Gao G., Ding S. (2019). A composite solid polymer electrolyte incorporating MnO_2_ nanosheets with reinforced mechanical properties and electrochemical stability for lithium metal batteries. J. Mater. Chem. A.

[32] Pereira-Pinheiro M. (2017). Studies of Interphase Formation between Biomolecules and Metal and Oxide Surfaces in Aqueous Environment. Master’s Thesis.

[33] Nguyen M.T., Sutton P., Palumbo A., Fischer M.G., Hua X., Gunkel I., Steiner U. (2021). Polymer-templated mesoporous lithium titanate microspheres for high-performance lithium batteries. Mater. Adv..

[34] Giannozzi P., Baroni S., Bonini N., Calandra M., Car R., Cavazzoni C., Ceresoli D., Chiarotti G.L., Cococcioni M., Dabo I. (2009). QUANTUM ESPRESSO: A modular and open-source software project for quantum simulations of materials. J. Phys. Condens. Matter.

[35] Perdew J.P., Burke K., Ernzerhof M. (1996). Generalized gradient approximation made simple. Phys. Rev. Lett..

[36] Corso A.D. (2014). Pseudopotentials periodic table: From H to Pu. Comput. Mater. Sci..

[37] Wildberger K., Lang P., Zeller R., Dederichs P.H. (1995). Fermi-Dirac distribution in ab initio Green’s-function calculations. Phys. Rev. B.

[38] The Materials Project Materials Data on Li4Ti5O12 by Materials Project. United States. https://www.osti.gov/dataexplorer/biblio/dataset/1284125.

[39] Calderín L., Karasiev V., Trickey S.B. (2017). Kubo–Greenwood electrical conductivity formulation and implementation for projector augmented wave datasets. Comput. Phys. Commun..

[40] Hoi B.D., Yarmohammadi M. (2018). The Kubo-Greenwood spin-dependent electrical conductivity of 2D transition-metal dichalcogenides and group-IV materials: A Green’s function study. J. Magn. Magn. Mater..

[41] Ureña Y.R.C., Lisboa-Filho P.N., Szardenings M., Gätjen L., Noeske P.-L.M., Rischka K. (2016). Formation and composition of adsorbates on hydrophobic carbon surfaces from aqueous laccase-maltodextrin mixture suspension. Appl. Surf. Sci..

[42] Gonçalves Dias L., Stamboroski S., Noeske P.L.M., Salz D., Rischka K., Pereira R., Mainardi M., Honorato M., Wiesing M., Bronze-Uhle E. (2020). New details of assembling bioactive films from dispersions of am-phiphilic molecules on titania surfaces. RSC Adv..

[43] Tian R., Alcala N., O’Neill S., Horvath D., Coelho J., Griffin A., Zhang Y., Nicolosi V., Colm O’Dwyer C., Coleman J. (2020). Quantifying the Effect of Electronic Conductivity on the Rate Performance of Nanocomposite Battery Electrodes. ACS Appl. Energy Mater..

[44] Mujica V., Kemp M., Roitberg A., Ratner M. (1996). Current-voltage characteristics of molecular wires: Eigenvalue staircase, Coulomb blockade, and rectification. J. Chem. Phys. A.

[45] Tian W., Datta S., Hong S., Reifenberger R., Henderson J.I., Kubiak C.P. (1998). Conductance spectra of molecular wires. J. Chem. Phys..

[46] Tomfohr J.K., Sankey O.F. (2002). Complex band structure, decay lengths, and Fermi level alignment in simple molecular electronic systems. Phys. Rev. B.

[47] Cui X.D., Primak A., Zarate X., Tomfohr J., Sankey O.F., Moore A.L., Moore T.A., Gust D., Harris G., Lindsay S.M. (2001). Reproducible Measurement of Single-Molecule Conductivity. Science.

[48] Beebe J.M., Engelkes V.B., Miller L.L., Frisbie C.D. (2002). Contact Resistance in Metal−Molecule−Metal Junctions Based on Aliphatic SAMs: Effects of Surface Linker and Metal Work Function. J. Am. Chem. Soc..

[49] Slowinski K., Fong H.K.Y., Majda M.J. (1999). Mercury−Mercury Tunneling Junctions. 1. Electron Tunneling Across Symmetric and Asymmetric Alkanethiolate Bilayers. J. Am. Chem. Soc..

[50] Cui X., Zarate X., Tomfohr J., Sankey O.F., Primak A., Moore A.L., Moore T.A., Gust D., Harris G., Lindsay S.M. (2001). Making electrical contacts to molecular monolayers. Nanotechnology.

[51] Haubner K., Murawski J., Olk P., Eng L.M., Ziegler C., Adolphi B., Jaehne E. (2010). The Route to Functional Graphene Oxide. ChemPhysChem.

[52] Acik M., Lee G., Mattevi C., Pirkle A., Wallace R., Chhowalla M., Cho K., Chabal Y. (2011). The Role of Oxygen during Thermal Reduction of Graphene Oxide Studied by Infrared Absorption Spectroscopy. J. Phys. Chem. C..

[53] Marsden A., Papageorgiou D., Vallés C., Liscio A., Palermo V., Bissett M., Young R., Kinloch I. (2018). Electrical percolation in graphene–polymer composites. 2D Mater..

[54] Melitz W., Shen J., Kummel A.C., Lee S. (2011). Kelvin probe force microscopy and its application. Surf. Sci. Rep..

[55] Salerno M., Dante S. (2018). Scanning Kelvin Probe Microscopy: Challenges and Perspectives towards Increased Application on Biomaterials and Biological Samples. Materials.

[56] Birkenhauer E., Neethirajan S. (2014). Surface Potential Measurement of Bacteria Using Kelvin Probe Force Microscopy. J. Vis. Exp..

[57] Chang K., Jun H., Hee Z., Hzeon J., Lee T., Won H., Kwak K., Park K., Zoung S. (2016). Effect of Amine-Based Organic Compounds on the Work-Function decrease of Graphene. J. Phys. Chem. C.

[58] Zhou Y., Fuentes-Hernandez C., Shim J., Meyer J., Giordano A., Li H., Winget P., Papadopoulos T., Cheun H., Kippelen B. (2012). A Universal Method to Produce Low-Work Function Electrodes. Science.

[59] Cheong K.Y., Tayeb I.A., Zhao F., Abdullah J.M. (2021). Review on resistive switching mechanisms of bio-organic thin film for non-volatile memory application. Nanotechnol. Rev..

[60] Chauhan A., Asylbekov E., Kespe S., Nirschl H. (2022). Influence of carbon binder domain on the performance of lithium-ion batteries: Impact of size and fractal dimension. Electrochem. Sci. Adv..

[61] Sun X., Radovanovic P.V., Cui B. (2016). ChemInform Abstract: Advances in Spinel Li4Ti5O12 Anode Materials for Lithium-Ion Batteries. ChemInform.

[62] Ahmad M., Han S.A., Tien D.H., Jung J., Seo Y. (2011). Local conductance measurement of graphene layer using conductive atomic force microscopy. J. Appl. Phys..

[63] Kim J.-B., Premkumar T., Giani O., Robin J.-J., Schue F., Geckeler K.E. (2008). Nanocomposites of poly(L-lysine) and single-walled carbon nanotubes. Polym. Int..

[64] Huang H.-H., De Silva K.K.H., Kumara G.R.A., Yoshimura M. (2018). Structural Evolution of Hydrothermally Derived Reduced Graphene Oxide. Sci. Rep..

[65] Liu H., Zhu Z., Huang J., He X., Chen Y., Zhang R., Lin R., Li Y., Yu S., Xing X. (2019). Elucidating the Limit of Li Insertion into the Spinel Li4Ti5O12. ACS Mater. Lett..

