# Supplementary material for: Application of Poly-L-Lysine for Tailoring Graphene Oxide Mediated Contact Formation between Lithium Titanium Oxide LTO Surfaces for Batteries
Source: Polymers (Basel). 2022 May 25;14(11):2150. doi: 10.3390/polym14112150 (PMC9182866; doi:10.3390/polym14112150)
Supplement: Supplementary file 1 [file polymers-14-02150-s001.zip › polymers-1701623-supplementary.pdf]

Application of Poly-L-lysine for Tailoring Graphene Oxide Mediated Contact Formation between Lithium Titanium Oxide LTO Surfaces for Batteries

Ignacio Borge-Durán [1,2+], Ilya Grinberg [1], José Roberto Vega-Baudrit [2,3], Tri Nguyen [4], Martha Pinheiro [5], Karsten Thiel [5], Paul-Ludwig Michael Noeske [5], Klaus Rischka [5] and Yendry Regina Corrales-Ureña [2,6,*]

1 Chemistry Department, Bar-Ilan University, Ramat-Gan 5290002, Israel; iborged@gmail.com (I.B.-D.); ilya.grinberg@biu.ac.il (I.G.)
2 National Laboratory of Nanotechnology LANOTEC, National Center of High Technology (CeNAT-CONARE), Pavas 1174-1200, San José, Costa Rica; jvegab@gmail.com
3 Laboratorio de Polímeros (POLIUNA), Universidad Nacional, Heredia 86-3000, Costa Rica
4 Adolphe Merkle Institute, University of Fribourg, Chemin des Verdiers 4, 1700 Fribourg, Switzerland; minhtri.nguyen@unifr.ch
5 Adhesive Bonding Technology and Surfaces, Fraunhofer Institute for Manufacturing Technology and
Advanced Materials IFAM, Wiener Straße 12, 28359 Bremen, Germany; m.pereira.pinheiro@tue.nl (M.P.); karsten.thiel@ifam.fraunhofer.de (K.T.); michael.noeske@ifam.fraunhofer.de (P.-L.M.N.); klaus.rischka@ifam.fraunhofer.de (K.R.)
6 Faculty of Production Engineering, University of Bremen, Am Fallturm 1, D-28359 Bremen, Germany
* Correspondence: *yendry386@hotmail.com, +borgedj@biu.ac.il

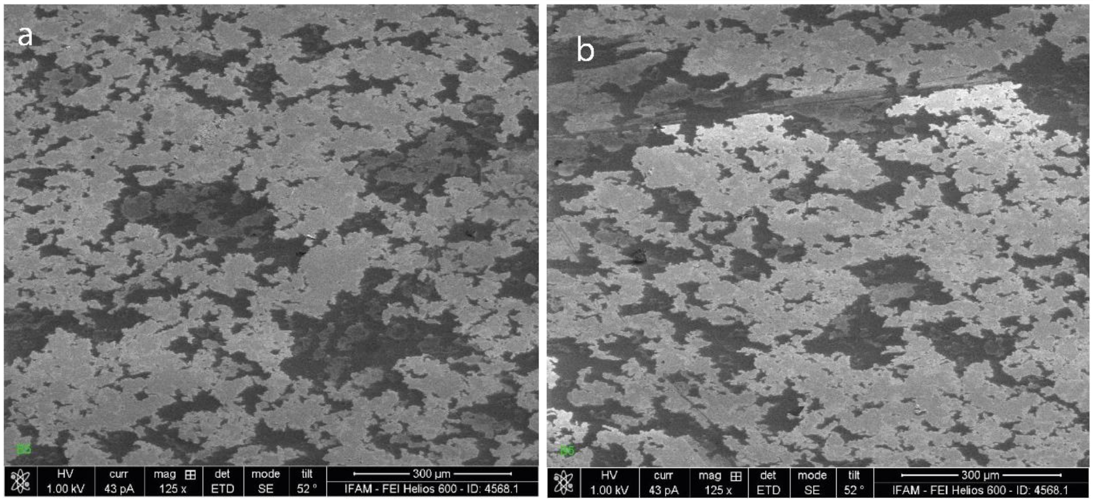

Supplementary Figure S1. Scanning Electron Microscopy (SEM) images recorded using detection of secondary electrons (SE-SEM) for TiO_2/PLL/rGO A) and B) with and without introduced scratches, respectively.
